# Cell Intrinsic Determinants of Alpha Herpesvirus Latency and Pathogenesis in the Nervous System

**DOI:** 10.3390/v15122284

**Published:** 2023-11-22

**Authors:** Stephanie Salazar, Khanh T. Y. Luong, Orkide O. Koyuncu

**Affiliations:** Department of Microbiology & Molecular Genetics, School of Medicine and Center for Virus Research, University of California, Irvine, CA 92697, USA; ssalaza3@uci.edu (S.S.); luongkt1@uci.edu (K.T.Y.L.)

**Keywords:** alpha herpesvirus, latency, reactivation, neurotropic virus, neuronal response

## Abstract

Alpha herpesvirus infections (α-HVs) are widespread, affecting more than 70% of the adult human population. Typically, the infections start in the mucosal epithelia, from which the viral particles invade the axons of the peripheral nervous system. In the nuclei of the peripheral ganglia, α-HVs establish a lifelong latency and eventually undergo multiple reactivation cycles. Upon reactivation, viral progeny can move into the nerves, back out toward the periphery where they entered the organism, or they can move toward the central nervous system (CNS). This latency–reactivation cycle is remarkably well controlled by the intricate actions of the intrinsic and innate immune responses of the host, and finely counteracted by the viral proteins in an effort to co-exist in the population. If this yin-yang- or Nash-equilibrium-like balance state is broken due to immune suppression or genetic mutations in the host response factors particularly in the CNS, or the presence of other pathogenic stimuli, α-HV reactivations might lead to life-threatening pathologies. In this review, we will summarize the molecular virus–host interactions starting from mucosal epithelia infections leading to the establishment of latency in the PNS and to possible CNS invasion by α-HVs, highlighting the pathologies associated with uncontrolled virus replication in the NS.

## 1. Introduction

Virus infections in peripheral tissues usually do not spread to the nervous system (NS) because of effective immune responses and multi-layer anatomical barriers. However, occasionally if these barriers are breached, infectious virus particles gain access to the NS via the bloodstream or by direct infection of nerves that innervate peripheral tissues [1]. Accidental nervous system infections by emerging viruses usually result in debilitating direct and immune-mediated pathology and mostly represent a dead-end for the host and the pathogen. However, few virus families, notably the alpha herpesviruses (α-HVs) such as herpes simplex virus (HSV), varicella-zoster virus (VZV), and pseudorabies virus (PRV) have evolved to enter the NS efficiently [2,3,4]. After entry, these virus particles move long distances by directed transport in the nerves to reach and establish infections in the peripheral ganglia [2,5]. Remarkably, α-HVs do not target the nervous system for a short period of time as in the case of rhabdoviruses (e.g., rabies virus), but rather for the life of the host [6,7]. In the lifetime of the infected host, α-HV infection can reactivate multiple times to spread to other hosts. This ingenious lifecycle is achieved by establishing a quiescent form of infection (i.e., latency) in the peripheral nervous system (PNS) after the primary infection of mucosal epithelia, with rare but often fatal central nervous system (CNS) pathology [8]. α-HV latency can be characterized by the episomal persistence of the viral genomes in the neuronal nuclei with limited transcription activity and lack of progeny production. This quiescent state can be reversed by the activation of stress signaling pathways leading to reactivation of the viral genome and initiation of productive replication [9].

Natural α-HV infections begin in a small number of epithelial cells at the mucosal surface. In response to virus infection, these cells manifest a local inflammatory response to clear the primary infection in the epithelial cells and to warn the surrounding cells of a potential danger via paracrine signaling [10,11]. Neurons innervating the infected epithelia are the primary target of the neurotropic α-HVs [12]. The axon termini of these neurons are not only the viral entry sites, but also are exposed to the inflammation environment initiated by the infected epithelia. In the PNS, axons extend long distances from the cell bodies to innervate distant tissues. Such a differentiated cellular architecture must have finely tuned long-distance communication between the axon termini and the cell bodies in the PNS ganglia to enable timely, effective, and properly controlled responses against infectious agents.

**Figure 1:**
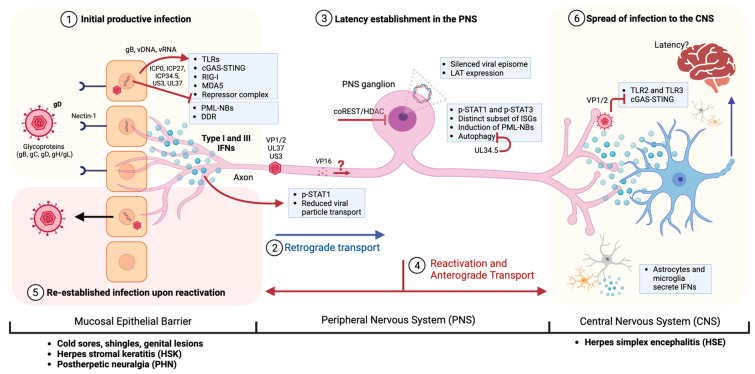
Model of HSV-1 infection and spread to the nervous system: (**1**) HSV-1 enters the mucosal epithelial cell and starts productive replication. Infection is detected by PRRs such as TLRs, cGAS-STING, RIG-I, and MDA-5, and receptor complexes, which induce the secretion of type I and III IFNs. Viral proteins such as ICP0, ICP27, ICP34.5, US3, and UL37 counteract antiviral sensing. (**2**) Once capsids enter axons of sensory neurons, outer tegument proteins are released in the cytoplasm while inner tegument proteins, such as UL36 (VP1/2), UL37, and US3, remain attached to the capsid. Within the axons, p-STAT1 is accumulated upon exposure to type I IFNs limiting retrograde viral particle transport. (**3**) HSV-1 genomes are maintained as circular chromatinized episomes during latency (except the LAT region). Activation of STAT1 and STAT3 leads to induction of a subset of ISGs in neurons. IFN exposure induces PML-NB formation in the neuronal nuclei. Autophagy restricts HSV-1 replication in neurons; however, UL34.5 inhibits Beclin-1-dependent autophagy. HDAC/CoREST/LSD1/REST repressor complex regulates latency and reactivation of viral genomes. (**4**) Reactivation of HSV-1 is triggered by stress signaling pathways, which alter silencing histone modifications, initiating the transcription of viral genes to produce infectious progeny. The new progeny can be transported anterogradely from the cell body toward the initial site of entry (**5**) triggering re-establishment of infection that can lead to cold sores, genital lesions, or shingles. Severe cases may result in oral or genital ulcerations, keratitis, or blindness via HSK, or post-herpetic neuralgia (PHN). (**6**) Upon reactivation, new progeny can also be transported anterogradely to the CNS, which is one synapse away. TLRs and cGAS-STING prompt an antiviral response in the CNS. TLR2 and TLR3 have demonstrated predominant protection against HSV-1 in the CNS. Astrocytes and neurons mostly rely on TLR3 for IFN production, with microglia activating the cGAS-STING pathway. VP1/2 targets the cGAS-STING in the CNS. It is still unclear whether α-HVs can establish latency in the CNS. References and abbreviations can be found throughout the text under corresponding sections.

How can α-HVs establish a lifelong latency in the host nervous system in the presence of active innate and adaptive immune responses without causing pathologies in otherwise healthy individuals? And how, in particular situations, do these infections lead to serious neuropathologies? To be able to understand this fascinating interplay and balance between the viral proteins, host neurons and the innate immune system, we will take a step-by-step approach dissecting the α-HV infection establishment in the NS after introducing the basics of α-HV infection and host response: (i) mucosal epithelial cell infection, (ii) neuroinvasion through the nerve fibers, (iii) latency establishment and reactivations in the PNS ganglia, and (iv) access to the CNS tissue (Figure 1). We will review findings on the intrinsic responses of each tissue to viral invasion together with viral countermeasures, and highlight the pathologies caused by α-HV infections due to specific mutations of the host genes or defects in the intrinsic and innate defense systems.

## 2. Basics of Intrinsic and Innate Immunity against Alpha Herpesvirus Infections

Alphaherpesvirinae is a subfamily that belongs to the *Herpesviridae* family, which infects a broad range of hosts [13]. The human α-HVs include herpes simplex virus 1 and 2 (HSV-1 and HSV-2), and varicella zoster virus (VZV) [14]. Well-studied veterinary α-HVs include pseudorabies virus (PRV), bovine herpesvirus 1 (BHV-1), and equine herpesviruses 1 and 4 (EHV-1 and EHV-4) [13,15]. PRV has been extensively used to study α-HV spread kinetics in neuronal culture and animal models. α-HVs are characterized by the large size of their double-stranded DNA. The dsDNA is encapsulated in a capsid, which is covered by a complex tegument layer and a membranous envelope. Most α-HV infections begin at the mucosal epithelium in the periphery such as the oral, genital, nasal, or oropharyngeal mucosa [16].

α-HV infections within the mucosal epithelia result in productive replication leading to the production of hundreds to thousands of progenies per infected cell [17,18]. Some of these newly made virus particles spread the infection to neighboring epithelial cells, fibroblasts, or nerve endings. Despite recent discoveries, there is a large knowledge gap between the connection of the mucosal epithelia and the PNS junction. Importantly, the virus–host interactions during the α-HV spread from epithelial cells to latency establishment within the peripheral ganglia have not yet been clearly understood.

Host intrinsic and innate responses play a vital role in protection against the spread of viral progeny and the induction of a systemic antiviral response against invading a-HV virions [10]. Both intrinsic and innate immune responses synergize to reduce or delay acute replication, albeit dependent on the cell type [19,20,21]. Intrinsic immunity provides an immediate and direct antiviral response against invasion of a pathogen, which is mediated via physical barriers and restriction factors. However, it lacks cell-to-cell communication and response amplification [22,23]. Intrinsic antiviral immunity is conferred by constitutively expressed host cell restriction factors that are encoded by almost all cell types [24,25]. To counteract viral invasion, these restriction factors directly and immediately act to subvert viral gene expression by inhibiting stages of the productive replication cycle, mostly followed by a self-degenerating process. Thus, understanding the host’s intrinsic immunity, and how it is induced and regulated in different cell types, is essential in understanding host cell protection and virus evasion mechanisms. 

Various cell types contribute to the innate immune response in both mucosal epithelia and the nervous system, providing additional support and protection during α-HV invasion [8,22,26]. Some of these cells include neutrophils, plasmacytoid dendritic cells (pDCs), and natural killer (NK) cells [27,28,29,30]. These cells play a vital role in the antiviral response partly due to their role in the synthesis and production of cytokines. Cytokines are small, secreted effector proteins that orchestrate the innate response against viral invasion. IFNs are cytokines produced in response to pathogenic invasion, and they activate a cascade of protein modification and transcription events through autocrine and paracrine signaling [31,32,33,34]. There are three categories of IFNs: type I, type II, and type III. Type I and type III IFNs are involved in intrinsic and innate immunity, whereas type II IFN (IFN-γ) is crucial in regulating innate and adaptive immune responses and will therefore not be discussed in the context of this review [26,27,35]. Type I and type III IFNs signal through distinct heterodimeric receptors that induce gene expression via the Janus kinase-signal transducers and activators of the transcription (JAK-STAT) pathway [36,37,38]. Upon activation of the JAK-STAT pathway, the IFN-stimulated gene factor 3 (ISGF3) complex, composed of phosphorylated STAT1 and STAT2 (p-STAT1 and p-STAT2) along with interferon regulatory receptor 9 (IRF9), is stimulated [31]. The ISGF3 complex enters the nucleus via the nuclear pores before binding to the IFN-stimulated response elements (ISREs) of IFN-stimulated genes (ISGs) [33]. Regardless of the overlapping downstream signaling pathways of type I and III IFNs, there are significant differences in the magnitude and kinetics of ISG induction between these two types of IFNs [39,40,41].

Type I IFNs (IFN-α and IFN-β) are essential to mediate a wide range of innate immune responses against viral infection and share a heterodimeric receptor, IFN alpha receptor (IFNAR), comprised of IFNAR1 and IFNAR2 subunits, which is ubiquitously expressed [31]. Early production of type I IFNs is crucial for initiating an antiviral response in infected and neighboring cells [42]. In vivo studies have shown that mice lacking type I IFN signaling have increased susceptibility to HSV-1 infection [43]. Furthermore, type I IFN deficiencies in humans have been linked to severe disease progression upon HSV-1 infection, such as herpes simplex encephalitis (HSE) [27,36,44,45,46].

In contrast to type I IFNs, type III IFN (IFN-λ) receptors are not ubiquitously expressed and have instead shown preferential expression on mucosal epithelial cells and immune cells such as neutrophils [31,38,41,47]. Type III IFNs consist of subsets IFN-λ 1-4 in humans while only IFN-λ2 and -λ3 are found in mice [31,48]. Type III IFN receptors consist of IL-10RB and IFNLR1 heterodimer. IL-10RB is broadly expressed and can be recognized by other members of the interleukin (IL) family whereas IFNLR1 has limited expression in epithelial cells and some immune cells [49,50]. Mucosal epithelial cells are a subset of epithelia that line the genital, respiratory, and gastrointestinal mucosal barriers [51]. Studies conducted on human intestinal epithelial cells suggest that the induction of and responsiveness to type III IFN are crucial aspects of an effective antiviral response [41]. Type III IFNs have demonstrated preferential antiviral response in mucosal epithelial cells as opposed to type I IFNs [52,53]. Type III IFN treatment of mucosal epithelia cells upon α-HV infection had a higher protective response when compared to type I IFN treatment [47,54,55]. Importantly, intravaginal pre-treatment of IFN-λ prior to HSV-2 infection in a murine model elicited a potent antiviral response [39,47,55]. Interestingly, the production of IFN-λ increases as cells polarize, and the quality of the IFN response is determined by the differentiation state of the mucosal epithelial cells [41,56].

## 3. Viral Infection Starts at the Mucosal Epithelia

HSV-1 entry into epithelial cells is achieved by receptor-mediated fusion of the viral envelope with the plasma membrane, requiring the concerted action of viral glycoproteins; gC, gB, gD, gH, and gL [57,58,59]. Cellular receptors nectin-1, a member of the tumor necrosis factor receptor family, and herpesvirus entry mediator (HVEM) serve as HSV-1 entry receptors [57,59,60,61]. The role of these receptors differs depending on the cell type and the route of infection. Nectin-1 is the major entry receptor for neuronal infection and is shown to be accumulated at cell–cell junctions [12,60,61,62,63]. gD on the viral membrane can bind to nectin-1, HVEM, or 3-O-sulfated heparan sulfate. Of those receptors, nectin-1 is the main entry receptor for neuronal and epithelial cells [64]. HVEM and nectin-1 as gD receptors and paired immunoglobulin-like type 2 receptor α (PILRA), myelin-associated glycoprotein (MAG), and myosin heavy chain 9 (MYH9) as gB receptors are also present in the adult human brain with increased expression in the hippocampus, which suggests underlying susceptibility of this brain region to HSV infection [65].

When bound to its receptor, gD undergoes conformational changes and activates the gH/gL heterodimer, which, in turn, activates gB, the principal fusogen that mediates the fusion of viral envelope and host cell membrane [66,67]. Upon entry, the viral nucleocapsid is transported to the nucleus via molecular motors. Only the inner tegument proteins (e.g., US3, UL37, and UL36) stay associated with the incoming nucleocapsids [3,68,69,70,71,72]. Outer tegument protein co-transport with nucleocapsids has rarely been observed during PRV and HSV post-entry transport [73]. Once the viral capsid and tegument proteins enter the cytoplasm, the tegument protein VP16, along with the host cell factors HCF-1 and Oct-1, travel to the nucleus independently, while the inner tegument proteins: UL36, UL37, and US3, remain attached to the capsid to facilitate transport on microtubules toward the cell nucleus [71,72,74]. Viral DNA is ejected into the nucleus, and viral genes are transcribed by host RNA polymerase II [75]. Productive α-HV infection follows a temporal cascade of protein synthesis, starting with immediate-early (IE) and early (E) gene expression, and followed by DNA replication and subsequent late (L) protein synthesis [7,17]. The relatively large genomes of α-HVs are not assembled into nucleosomes in the virions, but upon translocation into the nucleus, host histones are rapidly recruited onto viral genomes [76]. Through interactions with HCF-1 and Oct-1, incoming VP16 initiates the transcription of IE genes including infected cell proteins (ICPs): ICP0, ICP4, ICP22, ICP27, and ICP47 [77,78,79]. The IE proteins then participate in the transcription of early and late genes. Early proteins such as ICP8 and thymidine kinase (TK) carry out functions involved in viral DNA replication [17,80].

Once DNA replication occurs, late genes are expressed, followed by virus assembly [78,81]. Late genes can be subdivided into two classes: “leaky-late” or “true-late” genes. “Leaky-late” gene expression is amplified upon viral DNA replication with appreciable levels prior to replication while “strict-late” genes are expressed only upon viral DNA replication [82,83]. Viral DNA replication, capsid assembly, and DNA packaging occur in the nucleus [84]. Mature capsids bud from the nuclear membrane, acquire tegument proteins, and undergo secondary envelopment through the trans-Golgi network (TGN) [85,86]. Inner tegument proteins associate with the capsid first, while outer tegument proteins do so later [73,87,88]. The virions can egress from the infected cells to infect distant cells by using the canonical fusion machinery [66]. Alternatively, they can infect neighboring cells through cell–cell junctions using gE/gI heterodimers [89]. Besides viral proteins required for HSV-1 spread, Carmichael et al. recently discovered that host protein tyrosine phosphatase (PTP1B) is important for HSV-1 cell-to-cell spread [90].

During primary infection, the innate immune responses initiated at the infected mucosal surface are key in determining the efficiency of HSV-1 neuroinvasion and latency establishment. Innate immunity is activated upon the detection of viruses via pattern-recognition receptors (PRRs) [27,28]. PRRs recognize pathogen-associated molecular patterns (PAMPs), viral nucleic acids, and viral proteins, along with damage-associated molecular patterns (DAMPs) [91]. Cytosolic PRRs include the toll-like receptor (TLR) family, along with RNA sensors, RIG-I-like receptors (RLRs), and DNA sensors including cyclic GMP-AMP synthase (cGAS) [92,93,94,95,96]. Both RIG-I and MDA-5 are cytosolic RNA sensors that have been shown to be crucial factors for the detection of α-HV dsRNA [97,98]. Recently, it was found that HSV-1 viral kinase, Us3 specifically phosphorylates RIG-1 blocking downstream signaling that would otherwise lead to IFN production [99]. The function and regulation of RIG-I and MDA-5 have been reviewed during viral infection [94,95]. Here, we will focus on TLRs, the cGAS-STING pathway, and nuclear DNA sensors as host defense mechanisms that are targeted by multiple α-HV proteins, as one of the many functions of the α-HV IE genes is to circumvent host cell defenses to efficiently initiate viral gene expression.

### 3.1. Intrinsic Restriction Factors versus IE Proteins: TLRs

Toll-like receptors (TLRs) are PRRs that sense viral pathogen-associated molecular patterns, which initiate an intrinsic immune response and subsequent release of cytokines including IFNs [42]. Therein, TLRs play a pivotal role in HSV-1 infection and host response. There are ten functional TLRs encoded by the human genome (TLRs 1–10), whereas the mouse genome encodes for twelve functional TLRs: TLRs 1–9 and TLRs 11–13 [100]. Most studies, commonly utilizing genetically modified murine models, have investigated the PRR response to HSV-1 infection, demonstrating that TLR2, -3, -4, and -9 detect HSV glycoproteins [97,100,101,102]. It has been shown that TLR2, a plasma membrane receptor that recognizes HSV-1 glycoprotein B (gB), is activated upon α-HV infection, inducing a pro-inflammatory response [21]. Prior studies have highlighted the significance of the TLR2-mediated inflammatory response during HSV-1 infection, demonstrating the conferred protective effects of TLR2 and its cooperative role with other TLRs such as TLR4 and TLR9 [30,101,102].

Similarly, TLR4 recognizes the short-hairpin DNA during an HSV-2 infection, and this has been shown to increase the TLR4-dependent innate immune response in cervical epithelial cells [96,101]. Together, TLR2 and TLR4 launch a MyD88-dependent signaling cascade that induces macrophages and NK cells. However, ICP0, an IE protein of HSV-1, is capable of suppressing the TLR2-mediated innate immune response [103]. The role of TLR2 and TLR4 seems to be to contain viral infection as early as possible by activating cytokine expression in response to HSV infection.

On the other hand, TLR3 has been demonstrated to have a higher protective effect than TLR2 against α-HVs [46]. TLR3 is the only member of the TLR family that recruits toll/IL-1R domain-containing adaptor-inducing IFN-β (TRIF) and (TNF-associated factor) TRAF, signal transduction factors, instead of inducing a MyD88-dependent signaling pathway and inducing IFN synthesis upon HSV-1 recognition [46,100]. Recently, it has also been shown that TLR3 induces type I IFN in human fibroblasts and cortical neurons, restricting viral growth in vitro [97]. Studies have since shown substantial evidence of the protective role of TLR3 in HSV-1-mediated encephalitis, discussed in a later chapter in this review. HSV-1 evades TLR3 recognition by inhibiting its expression via the viral tegument protein kinase, US3, which inhibits the TNF receptor-associated factor-6, an adaptor of TLR signaling [104,105].

TLR9 is a complex DNA sensor with an essential role in IFN production, coordinating with other TLRs such as TLR2 to induce a pro-inflammatory immune response against α-HV infection [102]. During an HSV-1 infection, TLR9 initiates early and rapid production of type I IFNs and cytokine secretion via the interleukin-1 receptor-associated kinase 4 (IRAK-4) and MyD88-signaling pathways [21,106]. In mice, the main site of resistance against HSV-1 CNS disease seems to be TG because of the TLR2 and TLR9-mediated innate immune responses in the ganglia [107]. Interestingly, TLR2^−/−^ mice showed an increase in TLR9 expression but TLR9^−/−^ mice are not able to establish a successful immune response and are more susceptible to HSV-1-induced death [107]. In vivo, early type I IFN response seems to be dependent on TLR9 recognition of HSV-1 infection [108]. Additionally, HSV-1 infection in human cortical neurons resulted in the upregulation and activation of TLR9 upon type III IFN treatment [109]. TLR9 has also been shown to cooperate with DNA sensors such as the cyclic guanosine monophosphate–adenosine monophosphate (cGAMP) synthase (cGAS)—STING (The Stimulator of Interferon Genes) [110,111]. The activation of the cGAS-STING pathway leads to inhibitory signals that interfere with TLR9 activity. Further studies will elucidate the role of TLR9 and DNA sensors in combating α-HV infections in the NS.

### 3.2. cGAS-STING

Cytosolic α-HV DNA detection triggers the cGAS-STING pathway, leading to the expression of type I IFN-stimulated genes and the activation of innate immune responses [93]. cGAS produces cyclic GMP–AMP (cGAMP), which activates STING. Subsequently, STING dimerization is induced via TRIM56-mediated ubiquitination before being poli-ubiquitinated by TRIM32 in the Golgi complex. Tank binding kinase 1 (TBK1) binds and activates phosphorylation of STING, which then activates IRF3, a transcription factor, and induces transcription of type I IFNs [112]. STING deficiency in mice led to increased susceptibility to HSV-1 infection due to a lack of type I IFN induction [113].

Studies have demonstrated that STING is important for innate immunity against HSV-1 replication but is also essential for successful cell-specific viral replication [114]. Detection of viral DNA activates the cGAS/STING pathway to induce IFN production to combat viral replication, but several viral proteins have evolved to block this pathway: UL37 deamidates cGAS, ICP27 targets TBK1-mediated STING signaling, and UL41 binds to STING, blocking its translocation [115,116]. Moreover, the viral protein kinase US3 hyperphosphorylates IRF3, blocking its activation by TBK1 [117]. US3 also hyperphosphorylates and inactivates p65, a subunit of transcription factor NFĸB, which is crucial for IFN-β induction [28,118]. HSV-1 neurovirulence factor ICP34.5 binds and inhibits TBK1 [119]. Viral transcriptional activator VP16 blocks IRF3-CBP interaction [120]. A new pathway was discovered where HSV-1 induces expression of a miRNA targeting STING synthesis [121]. The interferon-inducible protein 16 (IFI16) is a nuclear DNA sensor recognizing the dsDNA of pathogens including that of α-HVs [122]. If IFI16 binding to the viral DNA is not blocked, this results in the induction of cytokines partly through the STING-TBK1-IRF3 signaling axis [114,123]. Therefore, ICP0, a viral E3 ubiquitin ligase and one of the first proteins to be expressed during HSV-1 infection, targets IFI16 for degradation. These findings emphasize that α-HVs encode multiple factors to ensure the success of its viral countermeasures.

In contrast to other PRRs, the cGAS-STING pathway is also capable of IFN-independent gene activation to mediate STING-dependent antiviral responses and induce a subset of ISGs [124,125]. The effect of the cGAS-STING pathway in CNS neurons will be discussed further in this review.

### 3.3. PML-NB (ND10)

Another set of nuclear proteins that are targeted by α-HVs is the components of the nuclear domains 10 (ND10), commonly known as promyelocytic leukemia protein nuclear bodies (PML-NB) [126]. PML-NBs are subnuclear organelles composed of several proteins that have the capacity to inhibit viral replication and transcription [127]. Therefore, these domains represent a major target for many DNA viruses, including α-HVs. Specifically, in HSV-1 infections, PML-NB is rapidly targeted and disrupted by ICP0. Immediately after the translocation of the viral genome into the host cell nucleus, HSV-1 genomes co-localize with PML-NB constituent proteins [128]. If ICP0 protein is not made, genomes continue to be “trapped” by these domains, which subsequently reduce the efficiency of viral transcription and replication.

ICP0 induces the degradation of cellular proteins such as PML-NBs and the centromeric repressive histone H3 variants [129,130,131,132] Other PML-NB constituent proteins include Sp100 (speckled protein of 100 kDa), hDaxx (human-death-domain-associated protein 6), and ATRX (alpha thalassemia/mental retardation syndrome X-linked), which have been demonstrated to limit the replication of ICP0-null mutants [131,133]. Since PML proteins act as the scaffold of PML-NB, they maintain and recruit other proteins to these nuclear domains. Therefore, PML loss leads to a dispersal of PML-NB-resident proteins and loss of intrinsic response [134,135]. Interestingly, studies have shown that exposure of cells to IFNs leads to an increase in the number of PML-NB bodies and an increase in PML proteins. PML ^+^/^+^ Hep2 cells treated with IFN led to a drastic reduction in HSV-1 virus yield while the antiviral effect was minimal in IFN-treated PML ^−^/^−^ Hep2 cells, indicating that PML-NBs contribute to IFN signaling resulting in a broad intrinsic antiviral activity [135,136,137]. Paradoxically, HSV-1 virus yield was substantially reduced in the absence of PML proteins in Hep2 cells at low-dose infections, suggesting a supportive role of PML-NB components for efficient virus replication [135].

Small ubiquitin-like modifiers (SUMO) are known to regulate and modify proteins of the PML-NB complex including PML and Sp100 [138]. ICP0 has been shown to directly degrade PML and Sp100 modified with SUMO proteins and work to counteract the intrinsic antiviral resistance of PML-NB and SUMO [139]. Conversely, SUMOylation has been shown to repress HSV-1 replication; loss of SUMOylation in cells enhances the permissiveness of HSV-1 ICP0-null mutants [139].

The inability of HSV-1 to initiate productive infection leads to the formation of latency-associated viral DNA-containing PML-NBs (vDCP-NBs), and this pattern is more likely to be initiated if the type I IFN pathway was activated prior to infection ([140]. The protein inhibitor of activated STAT1 (PIAS1) is a constituent PML-NB protein and part of the vDCP-NB complex that contributes to intrinsic antiviral response [141]. Both PIAS1 and PIAS2-α have been reported to localize to PML-NBs and to regulate PML SUMO modification, but only PIAS1 was shown to be a permanent constituent of PML-NBs ([141,142,143]. PIAS4 also plays a vital role in intrinsic immunity and is recruited independently of PML, a novel role that contrasts the known roles of PIAS proteins as suppressors of innate immunity to DNA virus infection [144]. 

PML-NB-associated proteins, ATRX and hDaxx, are involved in the formation of repressive chromatin modification on the HSV-1 genome. Although the exact role of ICP0 is not fully understood in this process, it has been found that hDaxx can act as a transcriptional repressor by interacting with histone deacetylases (HDACs) to induce the silencing of lytic viral promoters. Moreover, ATRX and hDaxx have been identified as critical regulators of replication-independent chromatin assembly [145]. Recent research has indicated that hDaxx and ATRX expression represses transcriptional activation, which is relieved by the expression of ICP0 [146].

Another strategy of host cells to repress viral gene expression involves chromatin repressor complexes and epigenetic modification mechanisms such as the HDAC/CoREST/LSD1/REST repressor complex, which acts as a critical component in regulating latency and reactivation [147]. ICP0 efficiently targets the components of the repressor complex and reverts its repressive activity on HSV-1 genomes [147]. ICP0 also promotes other chromatin modifications that stimulate productive infection [148,149]. ICP0-null mutants have impaired viral growth and replication deficiency in cell types such as fibroblasts and keratinocytes [150], mostly due to the presence of these nuclear host restriction factors providing a strong intrinsic anti-viral response to α-HV infections.

### 3.4. DNA Damage Response (DDR) Machinery

For successful DNA replication during productive infection, α-HV proteins must also counteract the host cell DNA damage responses (DDR). Genomes of incoming viral progeny are recognized by cellular DDR, and this activates DNA-damage-sensing kinases. These phosphatidylinositol 3-kinase-like serine/threonine protein kinases (PIKKs) include the DNA-dependent protein kinase (DNA-PK), ataxia telangiectasia mutated (ATM), and ATM and Rad3 related (ATR) along with the poly ADP-ribose polymerase (PARP) family [84,151]. PIKKs phosphorylate downstream factors and recruit repair factors or apoptosis in the case of damage [152]. The DNA-PK complex consists of the Ku70/Ku/80 heterodimer and promotes nonhomologous end joining (NHEJ) repair of DNA damage upon viral infection. Both ATM and ATR promote apoptosis in cells upon viral detection [153,154]. ATM is a chief controller of DDR and is activated by double-strand breaks [155]. ATM senses and promotes the repair of double-stranded DNA through both homologous recombination (HR) and single-strand annealing (SSA) [84,155]. ATR is activated through recognition of replicative stress during infection where it then induces DNA repair, cell cycle checkpoints, or apoptosis. ATR serves as a sensor for DNA replication fork collapse and replication complex uncoupling, making it essential for DNA replication [152]. 

Although DDRs induce an intrinsic antiviral response, HSV-1 manipulates DDR pathway components to support HSV-1 replication [156]. Prior studies have determined that ICP0 promotes the degradation of DDR factors, such as DNA-PK, preventing it from processing HSV DNA ends [84,152,157]. HSV-1 interferes with DDR machinery by promoting MDC1 and yH2AX accumulation at the viral genome along with interfering with crucial components for DNA repair such as p53 binding protein (53BP1) and BRCA1 [122]. In cortical neurons, HSV-1 has been shown to impair DNA repair by degrading KU complex components, impairing NHEJ activity [158]. Conversely, ATM and ATR have been shown to promote HSV-1 gene expression or promote replication in some instances during HSV-1 infection [156,159,160]. ATR is inhibited by HSV-1 ICP0 protein via the mislocalization of the ATR interacting protein during infection [161]. ICP0 targets histone ubiquitin ligases, RING (really interesting new gene) finger protein (RNF)-8, and RNF-168 downstream of ATM signaling to support viral DNA replication and efficient packaging of the viral genomes [162,163].

These studies provide insight into ways α-HVs can evade intrinsic antiviral response during productive infection in mucosal epithelia by targeting multiple sensors and repressive complexes. They also highlight the evolutionary importance of efficiently replicating the viral genome and yielding high numbers of progeny while combating a multi-faceted host immune response, some of which will invade the nervous system to establish a lifelong infection.

## 4. Recognition of Inflammation and Neuroinvasion of Peripheral Nerves

To establish life-long infections, α-HV particles egressing from mucosal epithelial cells must efficiently enter the peripheral nervous system (PNS) via the axonal termini of these neurons. The inflammatory cytokines secreted by the mucosal epithelial cells are sensed by the PNS axons even before the invasion by the viral progeny. How do neurons respond to this cytokine milieu? Can there be local antiviral responses in axons that are exposed to cytokines before they are exposed to infectious virus particles? Recent research shows that, in fact, these early communications between infected peripheral tissues and the nervous system play a pivotal role in determining the mode of infection in the neuronal ganglia and further affect the replication and reactivation dynamics [164,165,166].

### 4.1. Interferon Response: Type I versus Type III IFN

Neurons can sense and respond to immunostimulatory molecules and express type I IFNs themselves; albeit less than in mitotic cells [45,167,168]. The differential response to infection between different neuron types can influence the heterogeneity of HSV-1 latency as IFN-induced responses to HSV-1 in murine DRG-derived neurons seem to be reduced [168]. Interestingly, type I IFN treatment of neurons prior to infection with HSV-1 limited productive replication, promoted latency establishment, and even restricted reactivation of viral genomes in individual neurons [169,170]. In vitro cultures of TG have also demonstrated decreased viral replication upon dose-dependent type I IFN treatment prior to infection [171,172]. In the NS, type I IFNs have been shown to have a role in the early control of HSV-1 replication within trigeminal ganglia (TG), where they are sensed through the axons [172,173].

It is possible that during the early stages of infection, while the virus replication is controlled by the intrinsic and innate defenses at the mucosal surface, exposure of nerve termini to type I and III IFNs stimulate local responses in axons, independent of the distant cell bodies, which limit the entry and/or transport of α-HV particles [8,12,174]. In this first phase, neuronal cell bodies are possibly not engaged to reduce the risk of any unnecessary dangerous reaction. A previous study, using compartmentalized primary superior cervical neurons (SCGs) in tri-chambers showed that, when axons are exposed to IFN-β, STAT1 is phosphorylated, retained in axons, and initiates a non-canonical antiviral action [174]. The presence of phosphorylated STAT1 (pSTAT1) in axons interrupted retrograde HSV-1 and PRV capsid transport. The mechanism by which pSTAT1 in axons restricts the transport of herpes viral capsids is not clear. Accumulation of STATs in axons may trigger responses such as autophagy that would limit the number of capsids reaching the connected cell body. The number of virus particles reaching the neuronal nuclei significantly affects the mode of infection, as it was shown that there is a productive infection threshold (in the number of infecting viral particles), particularly when the infection is acquired through axons [175,176]. If the infection dose is below this threshold, viral genomes are immediately silenced in the neuronal nuclei before the lytic gene transcription is initiated, and latency is established. If the primary intrinsic and innate immune responses against virus infection are not effective, and the infection further spreads, innate and adaptive immune cells (e.g., NK and T cells) are activated. In this case, type II IFN, IFN-γ, produced by these cells not only affects infected epithelia but also alarms neuronal cell bodies through retrograde signaling about a potential viral invasion in the periphery. Indeed, in the presence of IFN-γ, authors showed that pSTAT1 is not retained in the axoplasm, but transported to the neuronal nucleus where it activates a global neuronal response including the canonical expression of numerous ISGs to shut down virus infection and in the worst case, to induce the apoptosis of the infected neuron [174]. This hypothesis was also supported by multiple other studies that have inferred that axonal pre-exposure to exogenous type I IFNs have an increased antiviral response against α-HV infection, essentially by priming the neuron for the upcoming viral invasion [11,174,177].

The potent antiviral role of type III IFNs, particularly in mucosal epithelial cells, has been expanded in recent years highlighting the tissue-protective potent antiviral action of these cytokines at the barrier surfaces. Because of these features, type III IFNs have been investigated for their potential to protect the nervous system against α-HV invasion. A recent study investigated the neuronal versus non-neuronal cell response to type III IFNs within the mucosa-NS junction using primary SCG neurons and fibroblasts. Interestingly, while type III IFN treatment led to STAT1 phosphorylation in both cell types, only fibroblasts showed STAT2 phosphorylation upon treatment [178]. This led to a differential ISG response in neurons characterized by the induction of a subset of SCGs at a lower magnitude. Type III IFN pre-treatment reduced PRV virus yield in both SCGs and Rat2s. However, whether type III IFNs can efficiently restrict HSV-1 latency establishment or reactivation remains to be elucidated. Danastas, et al. have recently shown that pre-treatment of isolated axons of DRG neurons grown in microfluidic chambers with IFN-λ impairs HSV-1 egress from axons. Interestingly, type I and type III IFNs were shown to induce only local STAT1 and STAT3 responses in axons, not in neuronal cell bodies. As expected, HSV-1 infection impaired the IFN signaling in neuronal cell bodies by limiting the translocation of pSTAT1 and pSTAT3 to the nucleus, further demonstrating viral evasion mechanisms involved in restricting IFN response [165]. These studies demonstrate the importance of the mucosal epithelial–neuronal cell junction, as paracrine cytokine signaling can ultimately affect productive infection and subsequent neuroinvasion.

### 4.2. The role of PML-NBs in Peripheral Neurons

A defining feature of the α-HV invasion of the PNS is the long-distance retrograde transport of nucleocapsids in axons separately from the outer tegument proteins. Partly due to the “shedding” of the outer tegument proteins (e.g., VP16) during axonal transport, incoming HSV-1 genomes cannot efficiently initiate the lytic gene transcription in neurons [7]. If the viral lytic gene transcription is not efficiently initiated, viral genomes are circularized and loaded with histone proteins that are further associated with silencing protein modifications and retained in the nuclei as heterochromatinized episomes [149,179,180]. During latency, viral gene expression is extremely limited, and viral DNA replication and progeny production is shut off. A hallmark of α-HV latency is the active transcription of the Latency Associated Transcript (LAT) region that not only produces a long non-coding RNA, but also several miRNAs that are crucial in maintaining the latency and reactivating the viral genome upon induction of appropriate stimuli ([181], see [182] for extensive review).

The fluorescent in situ hybridization (FISH)-mediated detection of HSV-1 genomes during latency in the mice trigeminal ganglia (TG) effectively demonstrated the role of PML-NBs in this process [183]. PML-NBs affected viral genome distribution and reduced LAT expression: HSV-1 genome localization with PML-NBs or centromeres was negatively correlated with LAT expression, indicating a role for these nuclear domains influencing HSV-1 latency and reactivation.

Interestingly, previous studies showed that sensory neurons lack these nuclear protein complexes in the absence of type I IFN, unlike non-neuronal cells in which HSV-1 replication was restricted efficiently [184]. However, it has been hypothesized that the presence of IFNs leads to the accumulation of PML-NBs in the neuronal nucleus. Indeed, this hypothesis was proven to be correct in a recent study: HSV-1 genomes were shown to colocalize with PML-NB during a latent infection only in the presence of type I IFN before the start of infection in an in vitro latency model [166]. Although PML-NBs may not be required for latency establishment, this study demonstrated that HSV-1 reactivations were restricted when PML-NBs were induced through type I and II IFN treatment [166]. Moreover, it has been suggested that neurons retain the memory of the immune response by retaining these nuclear domains [166]. The formation of PML-NB in response to cytokine sensing by neurons is critical for controlling HSV infection long after the acute phase, with significant effects on reactivation. Likewise, the presence of vDCP-NB-like structures in neurons of latently infected human TGs suggests that PML-NBs play a crucial role in controlling HSV-1 latency [140]. The role of PML-NBs within sensory neurons remains a field to further investigate in vitro and in animal models, particularly how the inflammation is sensed by the axons and how the signals are transmitted to the neuronal cell bodies to establish a nuclear state supporting a silenced α-HV infection that reactivates less frequently.

### 4.3. Autophagy

Autophagy is a highly conserved catabolic pathway that activates the cell death program as a survival mechanism. Studies have shown that autophagy plays a larger role in neurons as opposed to epithelial cells during HSV-1 infection [185,186,187]. A study performed by Yordy et al. (2012) determined that autophagy in epithelia does not succor local inflammatory response or antiviral defense during HSV-1 infection. Instead, autophagy was pertinent toward the sensory neuronal restriction of HSV-1 replication [168]. Further evidence suggests that autophagy provides a cell-intrinsic antiviral mechanism in the PNS against HSV-1 infection as shown in mouse DRG neurons [187]. Additionally, HSV infection and type I IFN (IFN-β) induced selective antiviral autophagy and autophagic clustering in peripheral neurons [168,188]. HSV-1 neurovirulence protein, ICP34.5, binds to Beclin-1, and inhibits Beclin-1-dependent autophagy [186]. Because of this viral protein, paracrine IFN signaling could not limit HSV-1 replication in TG neurons as it did vesicular stomatitis virus replication. Exogenous IFN-β is important for creating a functional antiviral state in TG neurons and restricting HSV infections [184,189]. ICP34.5 caused resistance to IFN-β signaling in neurons, partly due to its ability to bind to Beclin-1, supporting the idea that autophagy is a critical intrinsic antiviral response, particularly in the nervous system.

## 5. Latency Establishment in the PNS and Periodic Reactivations Leading to Pathologies

The intrinsic immunity, together with the extremely polarized and terminally differentiated state of peripheral neurons, plays an important role in the latency establishment of α-HVs. In addition to axonal infection leading to a repressive heterochromatin state and gene silencing, a lack of viral tegument proteins such as VP16 and ICP0 when viral genomes are ejected into the neuronal nuclei further challenges the initiation of the productive mode of infection [74,148,150]. As mentioned before, these outer tegument proteins are separated from the incoming capsids upon axonal entry, and it is still unclear whether they are retrogradely transported to the neuronal cell bodies or retained in axons [73,190].

In compartmented neuronal cultures, it was shown that if the tegument proteins are present in the neuronal cell bodies during axonal infections with PRV at a latency-establishing dose, viral genomes were able to escape from genome silencing [175]. If viral tegument proteins were not present at the time of axonal infection, activation of protein kinase A (PKA) in cell bodies enabled viral genomes to initiate productive infection via a cJun N-terminal kinase (JNK) dependent pathway [175]. Interestingly, this pathway took longer than the tegument-mediated escape from genome silencing. Moreover, in the presence of viral tegument proteins in the cell bodies, the activation of cellular kinases (PKA and JNK) was dispensable for the observed escape from the silencing phenomenon [164]. Although this study highlights the immediate action of tegument proteins on the activation of viral lytic gene promoters, it did not identify which tegument proteins are responsible for the rapid escape from genome silencing. As we know, ICP0 is responsible for the efficient onset of productive infection and may also trigger reactivation of latent viral genomes [30,130,191]. It is important to note that the absence of ICP0 leads to a repressive chromatin state driven by intrinsic and innate responses that are not counteracted [148,150,192]. Since VP16, together with the host cell factors HCF-1 and Oct-1, activate the transcription of IE genes (e.g., ICP0), it appears that the presence of this protein in the neuronal cell bodies (perhaps together with other tegument proteins) is one of the key determinants of the α-HV infection mode (productive versus latent).

A reactivation event differs from escape from silencing at least in two major aspects: the amount of silencing histone modifications on the viral genome and the lack of any tegument or structural viral proteins during latency [164,193]. Reactivation of latent α-HV genomes is usually triggered by stress such as physical trauma, sunburn, or fever (please see reviews [7,9,193,194]). When stress signaling pathways are activated, silencing histone modifications on viral genomes are altered and transcription of viral genes initiates following a biphasic program to yield infectious progeny [179,193]. A small portion of the newly made viral progeny in the neuronal soma can be sorted into the bifurcating axons of sensory neurons and are transported in the anterograde direction (the opposite direction relative to that during initial infection) toward the mucosal epithelia or to the brain [5,84,195] (Figure 1). Surprisingly, the threshold for reactivation of latently infected PNS neurons is high, such that reactivation does not occur in all the latently infected neurons [183,196,197]. Reactivation may be more likely to occur in neurons that contain more copies of the viral genome [196]. Even within a single neuronal nucleus, not all silenced genomes reactivate simultaneously [140,183,196,198]. Regulation of latency establishment, reactivation, and subsequent spread of infection is affected by cell-intrinsic, tissue-specific, and systemic factors, as well as viral proteins that are challenging to dissect and require complementary in vitro and in vivo studies. Currently, host and viral factors that affect the maintenance of latency and subsequent reactivation are not completely understood.

Reactivations result in typical cold sores, genital lesions, or shingles blisters [16,81,199]. Asymptomatic reactivation and shedding of HSV-1 [200] and VZV [201,202] are also known, but this phenomenon is more common in the case of HSV-2 reactivations [199,203]. However, α-HV reactivations may result in oral or genital ulcerations, keratitis or blindness (depending on the ganglia where latency is established), or post-herpetic neuralgia (PHN), and can even lead to encephalitis [199,204]. In this review, we will focus on the effect of α-HV reactivations on the NS.

### 5.1. Herpes Stromal Keratitis (HSK)

During the initial productive infection of the corneal epithelia, HSV-1 replicates and travels into the surrounding innervating neuronal axons where it establishes quiescence within the peripheral ganglia, specifically the trigeminal ganglia (TG) [205,206]. Sporadic reactivation of HSV-1 may occur within the host’s lifetime, especially in immune-deficient individuals, which leads to the re-infection of the primary site of infection. Recurrent infection of the corneal tissue causes scarring and vascularization and may lead to herpes stromal keratitis (HSK), the leading cause of corneal blindness in the United States [205]. During a productive infection, HSV-1 is recognized by the cell surface via PRRs, which initiate the innate immune response once more, leading to an influx of proinflammatory cytokines. At the cornea, HSV-1 recognition induces type I and type III IFN production [29,207]. IFNs provide protection from the viral transport of HSV-1 to the TG during acute ocular infection by inhibiting viral replication and reactivation [207,208,209]. Type I IFNs have shown a prominent protective response against viral infection in the cornea, preventing the systemic spread of HSV-1 to the TG in vitro and in vivo murine models [206,208]. Conrady et al. demonstrated that the TLR response to HSV-1 in the cornea was expendable. Instead, the DNA sensor, IFI16-mediated innate immunity was more significant in controlling the productive HSV-1 infection [210].

Recently, Miner, et al. showed that IFNLR1, which is expressed by the corneal epithelial cells, was inhibited by the action of antiretroviral drugs, AZT or TBK1 in human corneal explants resulting in increased HSV replication [211]. Apparently, type III IFNs induce a predominant protective antiviral response against HSV-1 infection in the cornea, which has also been demonstrated in other mucosal epithelial cells [47,52,54,55].

### 5.2. Post-Herpetic Neuralgia (PHN)

Similar to HSV, the reactivation of VZV leads to a self-limited dermatomal rash that is accompanied by a painful inflammation in the skin, commonly known as shingles or herpes zoster [212]. Dermatomal lesions can be treated with antivirals such as acyclovir and its derivatives [80]. However, in up to 50% of shingles patients, the pain persists even long after the resolution of the dermatome, resulting in a condition known as post-herpetic neuralgia (PHN) [213]. PHN is the most common long-term complication associated with α-HV reactivations and is commonly described as a burning or stabbing pain (in one side of the body) that can persist for months or even years, severely impacting the quality of life of patients suffering from this condition [214]. It was suggested that PHN is caused by VZV-induced neuroinflammation and axonal damage leading to hyperexcitability and spontaneous firing of PNS neurons [215]. Currently, it is widely accepted that two main factors significantly increase the risk of PHN: older age and immunosuppression [213]. Importantly, the available antiviral therapies that can be effective in the resolution of the dermatomal rash neither eliminate the risk of PHN nor show any effect on the established PHN [216]. Unfortunately, the commonly used clinical first-line treatments, including tricyclic antidepressants (TCA), lidocaine patches, non-TCA antidepressants, NMDA antagonists, ketamine, and Botox injections, rarely result in symptom resolution and do not offer long-lasting relief [213].

The molecular virus–host interactions and the neuro-immune response leading to PHN are far from understood. Recently, a mice model of PRV infection was proposed to study the mechanism of VZV-induced PHN, since PRV infection in mice induces a self-mutilating neuropathic itch that shows similarities to VZV-induced PHN [217]. In 1955, PRV infection of rats (non-natural animal host) was shown to induce spontaneous, intermittent discharge of nerve impulses over the preganglionic and postganglionic nerves of superior cervical ganglia (SCG) following ocular inoculation, leading to pruritus in the infected rats [218]. Interestingly, PRV infection of the natural host, adult pigs, does not lead to pruritus, instead results in the establishment of latency in the PNS ganglia, which typically is followed by sporadic reactivations similar to VZV or HSV infections in humans [219]. In contrast, PRV productively replicates in the PNS neurons of non-natural hosts following primary infection, latency is not established, and the infections almost always spread further to the CNS [220]. Importantly, the productive infection of PNS neurons triggers a specific inflammatory response initiating pruritus in non-natural hosts [220]. This difference in the pathogenesis of PRV between natural and non-natural hosts might give a hint as to why PHN is more likely to be a complication in VZV reactivations in the elderly and immunosuppressed: because well-controlled infections with VZV leading to latency in the PNS are established when the intrinsic responses of PNS neurons and the innate and adaptive immunity of the host strongly keep the infection in the NS in check. When these checkpoint responses weaken or are altered due to age or immunomodulatory conditions (similar to a non-natural host infection), VZV reactivations are more likely to lead to neuropathologies. Following these pioneering works, many in vitro and in vivo studies investigating PRV infections in different model systems helped identify several molecular aspects of the α-HV replication and spread in the nervous system [221,222,223,224,225]. Unraveling the molecular details of α-HV-induced pain and itch may lead to the development of innovative therapeutic strategies by determining viral-induced damage to the PNS, and understanding how this affects the communication with the CNS resulting in neurodegenerative processes.

## 6. Intrinsic and Innate Immune Response of the CNS to α-HV Infections

Although the pathologies associated with α-HV infections in the CNS are rare, the spread of an infectious virus to the CNS, either due to primary infection or upon reactivation, can occur through the synaptic connections of peripheral neurons or through olfactory receptor neurons (ORNs) [226,227]. Remarkably, α-HV particles can bidirectionally spread between synaptically connected neurons: from a pre-synaptic to a post-synaptic neuron (anterograde), or from a post-synaptic neuron to a pre-synaptic neuron (retrograde) [1,2]. Upon reactivation of latent α-HV genomes in the trigeminal ganglia, newly made progeny particles can be sorted anterogradely in the bifurcating axons of these pseudo unipolar neurons either arriving at the mucosa junction or at the CNS tissue that is one synapse away (Figure 1). It is not well documented whether virus particles travel to the CNS upon each reactivation cycle seeding the CNS neurons. Recent research supports the idea that viral particles spread to the CNS tissue more frequently than we used to think. Moreover, there is no specific barrier identified in these bifurcated axons that could prevent viral particle sorting into the CNS branch.

If viral particles find their way to the CNS neurons, they can initiate a productive infection in the brain resulting in encephalitis [228]. Herpes simplex encephalitis (HSE) is the most common cause of life-threatening sporadic encephalitis with most cases being due to reactivation of latent HSV infections [44]. There is also evidence from animal models that HSV can establish latency in the brain tissue. In a mouse model, HSV can reactivate from the brainstem immediately after tissue harvest, indicating that the brainstem can be a latency site for HSV in the CNS and potentially lead to a higher frequency of recurrent disease [229].

If the spread of α-HV particles into the CNS tissue is more frequent than previously anticipated, and if the brain can be a target for latency establishment and reactivations, why is HSE a dangerous but rare pathology? One explanation may be the attenuation of viral replication in neurons through virus–host co-evolution: the intrinsic immune responses of the terminally differentiated neurons limit transcriptional activation, translation, and replication of virus particles. From the viral side, this is strategically favorable since their life cycle has evolved to establish a lifelong persistence in the NS. Particularly in the CNS, the intrinsic immune response against α-HVs is more pronounced because of the immune-privileged state of this tissue. But immune surveillance does occur in the CNS: microglia and astrocytes are important resident immune cells responsible for immune surveillance in the CNS, and they express a wide range of toll-like receptors (TLRs) that help recognize pathogen-associated motifs (PAMPs) [230,231,232].

### 6.1. Toll-like Receptor Signaling Pathway (TLR2, TLR9, and TLR3)

TLR2 is an extracellular membrane TLR that senses HSV entry glycoproteins such as gB, gD, gH, and gL and induces antiviral innate immune response [91]. Upon activation, TLR2 dimerizes with TLR1, TLR2, or TLR6, which then activates NF-κB and IRF3, and upregulates IFN and cytokines. Differential activation of TLR2 occurs in different cell types: TLR2 homodimer induces IFN-β in neurons and IFN-α in astrocytes. These IFNs subsequently activate ISGs including viperin, Ch25H, OAS2, latent RNase (RNase L), protein kinase R (PKR), and IFIT1 [233]. TLR2 and TLR9 have been shown to act synergistically to upregulate an early cytokine and cellular response to restrict HSV-2 viral load in the brain [102]. Mice with TLR2^−/−^ and TLR9^−/−^ have higher viral loads and show no induction of the ISG CXCL9 in the brain. In another report, TLR2-mediated inflammatory cytokine response was associated with lethal encephalitis upon HSV-1 infection in mouse neonates. In this study, wild-type mice demonstrated elevated levels of IL-6 and monocyte chemoattractant protein-1 (MCP-1) in the brain as compared to TLR2^−/−^ mice, leading to brain hemorrhage and death [91].

TLR3 is an endosomal TLR that recognizes dsRNA and is an important intracellular sensor of HSV that signals the intrinsic immune response [97]. TLR3 deficiency can impair the production of Type I and Type III IFNs, leading to higher susceptibility to HSE. Interestingly, TLR3 defects in the CNS affect IFN production differently from those in the PNS [234]. A TLR3 defect is partial as it does not entirely abolish the induction of IFN-β and -λ in dermal fibroblasts, demonstrating a redundant mechanism—and therefore importance—of IFN induction for antiviral response outside the CNS [97,234]. The effect of TLR3 deficiency in the CNS is similar to that found in the periphery during HSV-2 infection: Astrocytes are known to express a high level of TLR3. Astrocytes with TLR3^−/−^ have impaired type I IFN production and exhibit elevated permissiveness to HSV-2 infection both in vivo and in vitro [235]. Reinert et al. suggest that astrocytes play an important role in sensing HSV entry into the CNS and induce a type I IFN response to restrict the virus (2012). Studies using CNS-resident cells derived from human induced pluripotent stem cells (hiPSC) showed that TLR3 expression in neurons and oligodendrocytes is essential in controlling HSV infection in the CNS [236]. Cells deficient in TLR3 can be rescued by exogenous IFN-α/β, but not by IFN-λ [237]. Furthermore, hiPSC-derived cortical neurons possess both a TLR3-dependent, constitutive immunity to HSV that antagonizes HSV-1 infection—in the absence of Type I IFN preconditioning—and an inducible resistance to HSV-1 infection that is different from hiPSC-derived TG, which lacks constitutive resistance but possesses an inducible resistance to HSV-1 infection [237,238]. The constitutive resistance of cortical neurons via TLR3-dependent IFN production may contribute to the prevention of HSV spread in the CNS and reduce HSE susceptibility in those with wild-type TLR3. Recently, Lafaille et al. discovered that mutation of human SNORA31 renders hPSC-derived cortical neurons susceptible to HSV-1 and exogenous IFN-β renders SNORA31- and TLR3- but not STAT1-mutated neurons resistant to HSV-1 [239]. This also hints that STAT-1 from the JAK/STAT pathway is crucial in relaying Type I IFN signaling in the CNS.

### 6.2. cGAS/STING Pathway

The role of the cGAS/STING pathway in sensing HSV infection in the CNS has only recently been investigated. The cGAS/STING pathway is another important sensor of microbial infection that initiates intrinsic immune responses against the invader by detecting dsDNA in the cytoplasm [92]. This pathway activates the production of Type I IFN to elicit the antiviral response [113]. Whereas astrocytes and neurons are dependent on TLR3 for the initiation of IFN production, microglia can sense HSV-1 infection through the cGAS/STING pathway [240]. The mechanism of response to HSV appears to depend on the infectious viral dose: At a high viral dose (multiplicity of infection -MOI of 15), infected hiPSC-derived microglia induced cGAS-dependent apoptosis, which consequently downmodulates local immune response, whereas at a lower viral dose (MOI of 5), cGAS activation induced Type I IFN production and antiviral activity [241].

This dose-dependent phenotype can partly be explained by synchronous versus asynchronous infection during 15 and 5 MOI infections, respectively. In the latter dose, paracrine signaling from the primarily infected neurons might initiate an antiviral state in the surrounding cells to further limit viral replication. Alternatively, the number of viral proteins present at the time of infection detected by the host cell determines the subsequent response: α-HVs encode and (pack) viral proteins that help evade the intrinsic immune effectors of the host cell. The cGAS/STING pathway is tightly regulated by a post-translational modification that also includes ubiquitination [242,243]. Specifically, the K27- and K63-linked polyubiquitination of STING has been shown to promote downstream recruitment of TBK1 and activation of IRF3 [92]. The deconjugation of polyubiquitin chains from STING prevents TBK1 recruitment and thereby negatively regulates the cellular antiviral response [244]. Bodda et al. discovered that HSV-1 viral protein VP1/2 can de-ubiquitinate STING through the digestion of the K63-linked ubiquitin chains, which thereby inhibits downstream signaling and type I IFN expression in response to HSV-1 infection [245]. VP1/2 (i.e., UL36) is the largest tegument protein of HSV. It is one of the few tegument proteins that stay bound to the incoming nucleocapsids upon viral entry into a neuron. The outer tegument proteins dissociate into the cytoplasm, but the inner tegument proteins (e.g., VP1/2, UL36, and Us3) travel together with the nucleocapsids on their way to the nucleus [190]. The main function of VP1/2 (together with UL37) during viral neuroinvasion is the recruitment of the motor protein, dynein, onto the nucleocapsids to ensure the transport of the nucleocapsids on microtubule tracks until the viral genome is delivered into the neuronal nucleus [72]. It is tempting to speculate that the VP1/2 associated with the incoming nucleocapsids target STING even before the viral gene expression is initiated in the nucleus. The interaction partner of VP1/2, UL37, has been shown to be essential in the nervous system invasion of both PRV and HSV-1 in animal models. Interestingly, mutations in the R2 region of UL37 did not affect viral replication in epithelial cells but blocked nervous system infection [246].

Type I and III IFNs are important cytokines that control HSV infections in the brain, but little is known about the ISGs, the effectors of the IFN pathway that combat viral infection in the CNS, and whether their expression is similar to or different from the epithelial cells. [109,235]. Comparative analyses of tissue-specific responses to infection and cytokines will help better understand the regulation of α-HV replication and viral pathogenesis in the NS.

## 7. Concluding Remarks

Intrinsic responses in non-neuronal tissues and the nervous system are often conserved, yet the “default” α-HV infection mode is dramatically different in epithelia versus the nervous system. The latent mode of infection almost seems to be tailored to the highly polarized morphology and the terminally differentiated biology of the peripheral neurons. Despite the recent advances in the molecular and systemic dissection of α-HV neuronal infections, we are far from deciphering how the initial peripheral productive infection and inflammation are sensed by the nervous system, influencing the establishment and control of viral persistence for the life of the host. Importantly, the nervous system mainly relies on intrinsic and innate immune responses to avoid extensive inflammation and tissue damage. The molecular sensors that are present in the nerve fibers provide a first-line defense against viral invaders and summon the ganglia and immune cells that confer further resistance. These initial virus–host–immune system interactions and long-distance inter-tissue communications are pivotal in asymptomatic reactivations turning into life-threatening pathologies. Intriguingly, even in subclinical cases, there are host-to-host variations in the extent and the frequency of α-HV reactivations and shedding, which almost always occurs in a seropositive state. As we learned from non-natural host infections with PRV, when the initial host response is inadequate (or tailored to the species-specific pathogen), α-HV proteins alter neurophysiology leading to synchronous continuous firing of neurons, eventually spreading the infection to the brain, and killing the host. It is fascinating that in the natural hosts, virus–host–immune system interactions are so finely equilibrated that infections lead to latency establishment in the nervous system with no apparent pathologies even when viral particles reach the CNS neurons during reactivations.

Many molecular details of virus–host interactions are missing, particularly regarding the communication of peripheral tissues with the nervous system, as well as local and global intrinsic responses of neurons to viral infection and inflammation. Does the exposure of nerves to infected epithelial cytokines induce a canonical signaling pathway leading to gene expression in the neuronal nuclei similar to when the neuronal cell bodies are exposed to cytokines in the ganglia, or does it induce differential signaling leading to alternative antiviral mechanisms? Does it stay local in the axons, or is it always conveyed to the neuronal nuclei? Another important question is whether human α-HVs can establish authentic latency (with occasional reactivations) in the CNS neurons. If so, are the silencing or reactivation dynamics different from the PNS neurons? More importantly, what prevents the viral genomes from lytic replication in the brain, causing HSE upon reactivations? Answering these questions requires further molecular investigations of virus–host interactions complementing the animal models of α-HV latency and reactivation.

## Data Availability

Not applicable.
